# Small Molecules and Immunotherapy Agents for Enhancing Radiotherapy in Glioblastoma

**DOI:** 10.3390/biomedicines10071763

**Published:** 2022-07-21

**Authors:** Jennifer K. Matsui, Haley K. Perlow, Alex R. Ritter, Rituraj Upadhyay, Raju R. Raval, Evan M. Thomas, Sasha J. Beyer, Clement Pillainayagam, Justin Goranovich, Shirley Ong, Pierre Giglio, Joshua D. Palmer

**Affiliations:** 1 College of Medicine, The Ohio State University, Columbus, OH 43210, USA; jennifer.matsui@osumc.edu; 2 Department of Radiation Oncology, The Ohio State University Wexner Medical Center, Columbus, OH 43210, USA; haley.perlow@osumc.edu (H.K.P.); alex.ritter@osumc.edu (A.R.R.); rituraj.upadhyay@osumc.edu (R.U.); raju.raval@osumc.edu (R.R.R.); evan.thomas@osumc.edu (E.M.T.); sasha.beyer@osumc.edu (S.J.B.); 3 Department of Neuro-Oncology, The Ohio State University Wexner Medical Center, Columbus, OH 43210, USA; clement.pillainayagam@osumc.edu (C.P.); justin.goranovich@osumc.edu (J.G.); shirley.ong@osumc.edu (S.O.); pierre.giglio@osumc.edu (P.G.)

**Keywords:** glioblastoma, radioresistance, radiosensitizer, glioma stem cell, tumor hypoxia

## Abstract

Glioblastoma (GBM) is an aggressive primary brain tumor that is associated with a poor prognosis and quality of life. The standard of care has changed minimally over the past two decades and currently consists of surgery followed by radiotherapy (RT), concomitant and adjuvant temozolomide, and tumor treating fields (TTF). Factors such as tumor hypoxia and the presence of glioma stem cells contribute to the radioresistant nature of GBM. In this review, we discuss the current treatment modalities, mechanisms of radioresistance, and studies that have evaluated promising radiosensitizers. Specifically, we highlight small molecules and immunotherapy agents that have been studied in conjunction with RT in clinical trials. Recent preclinical studies involving GBM radiosensitizers are also discussed.

## 1. Introduction

Glioblastoma (GBM) is the most common malignant primary brain tumor with a dismal five-year relative survival rate of 6.8% [1]. Despite decades of preclinical and clinical studies, survival and quality of life outcomes have not substantially improved. Currently, the standard treatment approach consists of surgery, radiation treatment (RT), temozolomide (TMZ), and tumor-treating fields (TTF) [2,3]. Numerous clinical trials have attempted to further improve survival outcomes but have generally been disappointing. Although trial results have been underwhelming, there is a greater understanding of the mechanisms driving treatment resistance. Specifically, researchers have uncovered ways the GBM tumor microenvironment (TME) promotes tumorigenesis, disease progression, and radioresistance [4]. More recently, small molecules and immunotherapy agents have been designed to enhance RT efficacy by targeting GBM tumor hypoxia and metabolic reprogramming. In this review article, we aim to highlight studies that have evaluated small molecules and immunotherapy agents as radiosensitizers in GBM patients.

## 2. Modern Treatment Strategies for Glioblastoma

GBM is among the most challenging cancers to treat because of tumor location, tumor heterogeneity, and the infiltrating growth pattern [4]. For patients with good performance status (Karnofsky performance status ≥60), the median overall survival (OS) rate is approximately 14 months [2]. Standard treatment modalities are discussed below.

### 2.1. Surgical Resection

Numerous studies have found aggressive, maximal tumor resection is associated with better survival outcomes [5,6]. Unfortunately, complete resection is often not possible due to the diffuse nature of the disease, where the tumor is frequently located in or near the eloquent cortex [7]. Although new surgical techniques have been developed (e.g., neuronavigation, fluorescence, intraoperative imaging), they have failed to significantly improve the prognosis for GBM patients [8].

### 2.2. Chemotherapy

**Figure 1:**
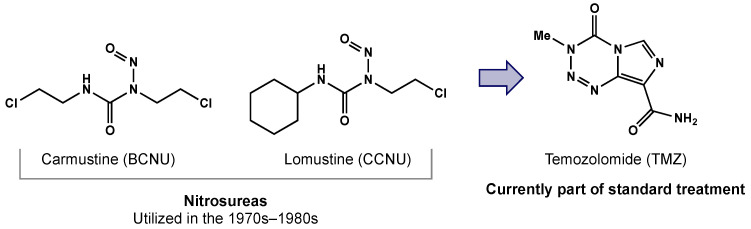
TMZ replacing nitrosoureas as the standard chemotherapy agent for GBM.

TMZ has replaced nitrosoureas (e.g., carmustine, lomustine) as the standard chemotherapy for patients with GBM (Figure 1) [9]. TMZ is an alkylating agent that preferentially methylates DNA at the N7 and O6 positions of guanine and the N3 position of adenine [10]. The methyl adducts result in cycles of mismatch repair with eventual strand breaks and ultimately cell cycle arrest at the G2/M phase [11]. Patients with methylation of the MGMT promoter experienced a survival benefit compared to patients who did not have a methylated promoter [12]. This phenomenon is attributed to patients with hypermethylation having decreased expression of the DNA repair enzyme [12]. Lower levels of MGMT therefore prevent mismatch repair and enhance the efficacy of TMZ.

### 2.3. Radiotherapy

The current standard treatment for newly diagnosed GBM is based on a randomized phase III trial led by the European Organization for Research and Treatment of Cancer (EORTC) 26981-22981/National Cancer Institute of Canada Clinical Trials Group (NCIC CTG). This study found RT plus concomitant and adjuvant TMZ led to improved median progression-free survival (PFS; 6.9 vs. 5.0 months) and median OS (14.6 vs. 12.1 months) compared to RT alone [2]. Based on these findings, GBM patients receive post-operative radiotherapy and concomitant TMZ (75 mg/m^2^ of body-surface area per day) [13]. The radiation treatment is typically administered over six weeks via 3-D conformal or intensity-modulated radiotherapy (IMRT). A standard treatment schedule is 2 Gy per day given Monday through Friday for a total dose of 60 Gy. Following the radiation course, maintenance TMZ (150–200 mg/m^2^) is given over 5 days every 28 days for 6 cycles. More recently, Stupp et al. evaluated the safety and efficacy of TTF (low-intensity, intermediate-frequency alternating electric fields) [14] following chemoradiation [3]. Median PFS and OS were significantly prolonged in the TMZ plus TTF vs. TMZ alone [3,15].

Hypofractionation is an alternative strategy where RT is administered in larger doses per fraction over fewer fractions. Recently, radiation oncologists have explored hypofractionation as a shorter, more convenient alternative, particularly in the elderly and/or frail patient population [16,17,18,19,20,21,22]. Preclinical data have suggested high dose per fraction results in a superior immunologic response that creates an abscopal effect [23,24]. Studies have shown hypofractionation has a stimulating effect on the anti-tumor immune response by inducing tumor cell death, normalizing irregular tumor vasculature, and releasing tumor-associated antigens [25,26]. Hypofractionated RT is currently being explored in combination with immunotherapy [27]. Other active areas of RT research include image-guided radiotherapy (IGRT) [28] and particle therapy [29,30,31].

## 3. Mechanisms of Radiation-Induced Cancer Cell Death

Radiotherapy has been a cornerstone treatment modality for treating GBM. Radiation-induced tumor cell killing can either occur via direct DNA damage or indirectly through the generation of radicals (e.g., peroxyl, hydroxyl radicals) [32]. Radiation can create DNA base lesions (e.g., 8-oxo-guanaine, formamidopyrimidines) and single- or double-strand DNA breaks. By definition, two or more lesions found within two helical turns are defined as a “clustered lesion” [33] while two strands of DNA phosphodiester backbone breaks within 10 base pairs is a double-strand break [34]. A dose of 1 Gy is estimated to produce around 3000 damaged bases, 1000 single-strand breaks, and 40 double-strand breaks [35]. Double-strand breaks are particularly difficult to repair and lead to greater cancer cell death [36].

The Bremsstrahlung process refers to inelastic interactions between an electron and nucleus that releases a photon, and this produces X-rays in linear accelerators used for radiation. Photon interactions with X-rays can be categorized into diagnostic (energy of 20–150 kV and used for imaging), superficial (50–200 kV, used for skin), orthovoltage (200–500 kV, used for skin and ribs), and megavoltage (1–25 MV, used for deep tissues). Irradiation has the ability to inhibit cancer cell proliferation through the stimulation of cell death mechanisms (e.g., apoptosis, necrosis, senescence) or by damaging cell membranes and organelles, impairing important signal transduction pathways [37,38].

## 4. Radioresistance

Despite advances in the field of radiation oncology (e.g., 2-dimensional WBRT to 3-D conformal RT and IMRT), radioresistance remains a challenging aspect of treating GBM patients. Radiation can induce DNA single- or double-strand breaks, leading to a decrease in radiosensitive tumor cell proliferation. DNA repair mechanisms are then activated, but cell death ultimately results if the damage is irreparable. A small subset of cells can evade apoptosis and instead become overactive. These surviving cells have alterations in tumor suppressor and oncogene expression that lead to radioresistance [39]. Furthermore, the tumor microenvironment (TME), tumor hypoxia, and glioma stem cells (GSCs) are other factors contributing to treatment failure [4]. Understanding these mechanisms has led to the rational development of drug inhibitors.

### 4.1. Glioma Stem Cells

Cancer stem cells (CSCs) are located within tumor masses and are able to self-renew and differentiate into various tumor cell types [40]. Researchers hypothesize these CSCs have the ability to generate the heterogeneous cell population seen in tumors. CSCs exhibit greater radioresistance due to their DNA-repair mechanisms, ROS scavenging systems, and self-renewal capabilities [41]. In GBM, these cells are known as glioma stem cells (GSCs) and have the ability to propagate as RT-resistant cells [41,42]. GSCs are able to express markers that regulate various pathways, telomerase activity, transporter proteins, cytokine secretion, and pro-angiogenic factors [43]. Glioma initiating cells (GICs) are a subpopulation of GSCs in the tumor microenvironment and play a key role in tumor heterogeneity. The heterogeneity causes challenges in treatment when there are variations in gene status (e.g., IDH, MGMT) [44].

### 4.2. Hypoxia

In the 1950s, Thomlinson and Gray published a landmark paper that suggested hypoxia may play a key role in tumor radioresistance [45]. Subsequent studies have supported this notion; it is estimated a radiation dose needs to be three times higher for hypoxic regions to induce the same DNA damage as in normal oxygenated regions [46]. This phenomenon is explained through the “oxygen fixation hypothesis,” where radicals are produced through ionizing radiation that interacts with neighboring oxygen, causing the formation of reactive oxygen species (ROSs) [47]. The resulting radical species then irreversibly damages DNA.

Key studies have suggested the TME in GBM plays a key role in the development of tumor hypoxia [48]. The TME is composed of stromal cells, signaling molecules, immune cells, and the surrounding extracellular matrix [49]. This complex matrix of cells creates pockets of hypoxia and acidosis via “microvascular hyperplasia” where rapidly dividing endothelial cells form microaggregates of sprouting vessels [50]. During this rapid growth, there is a complex interplay between cells and the extracellular environment that creates structural abnormalities (e.g., incomplete or absent basement membranes, irregular architecture) [51]. These abnormalities cause irregular blood flow, allowing tumor cells to invade beyond the diffusion distance of oxygen within the tissue. To supply oxygen to the tumor cells, angiogenesis is mediated by hypoxia-inducible factors (HIFs) to create new capillary systems. These inefficient capillary systems maintained by the tumor create an oxygen gradient.

## 5. Radiosensitizers

The rationale behind combining radiation and chemotherapy originates from the Steel paradigm [52]. Steel et al. proposed that synergy is driven by (1) spatial cooperation, (2) toxicity independence, (3) protection of normal tissues, and (4) enhancement of tumor response. The enhancement effect can be driven by inhibiting radiation-induced damage, reoxygenation following treatment, and/or improved drug access following RT.

Early studies demonstrated some chemotherapeutics such as cisplatin have the ability to sensitize tumor cells to RT, leading to greater radiation efficacy [53]. More recently, radiosensitizers have been developed that work through a variety of mechanisms: (1) Suppression of intracellular thiols or other radioprotective substances, (2) radiation-induced formation of cytotoxic substances via radiolysis of the sensitizer, (3) inhibition of the post-radiation cellular repair processes, (4) structural incorporation of thymine analogues into intracellular DNA, and (5) oxygen mimetic sensitizers [54,55].

Although other disease sites have found success with radiosensitizers, GBM has been particularly challenging due to its anatomic location (e.g., located beyond the blood–brain barrier), cell heterogeneity (e.g., cancer stem cells, tumor microtubes), and increased proliferation rate [56]. To date, TMZ is the most effective and widely used radiosensitizer in the treatment of GBM. TMZ increases the number of RT-induced double-strand DNA breaks as a result of a decrease in DNA repair capacity [57,58]. This review will focus on other small molecule and immunotherapy agents that have shown preclinical promise. Additionally, we will discuss relevant clinical trial findings.

### 5.1. Pyrmidine Analogues

**Figure 2:**
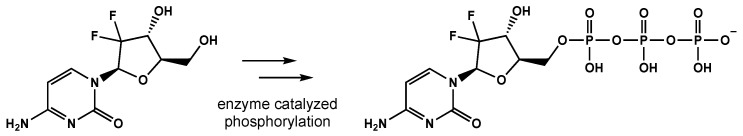
The conversion of gemcitabine to gemcitabine-5′-triphosphate before being incorporated into DNA and RNA, eventually leading to strand termination.

Gemcitabine is a difluoro-pyrimidine analog that is phosphorylated and incorporated into the DNA and RNA of cancer cells, leading to chain termination (Figure 2) [59]. The radiosensitizing effects of gemcitabine result from the depletion of phosphorylated deoxynucleotides and cell-cycle redistribution into the S-phase [60,61,62]. To date, gemcitabine has demonstrated activity in breast, ovarian, non-small cell lung, pancreatic, and bladder cancers [63].

In vitro studies have determined the gemcitabine administration schedule is essential for maximal radiosensitzation. Gemcitabine achieved radiosensitization with long exposure (24 h) to low gemcitabine concentrations or brief treatments with increased concentrations [64]. Maraveyas and coworkers conducted a phase I study in brain metastases patients evaluating the maximum tolerated dose of concomitant gemcitabine and RT [65]. A phase I study then evaluated gemcitabine with concomitant RT in newly diagnosed GBM patients [66]. In this study, gemcitabine was delivered at 10 mg/m^2^/min on a weekly basis for 6 weeks 24 to 72 h prior to concomitant RT (60 Gy in 30 fractions) with the identification of dose-limiting toxicity and maximum tolerated dose as the primary end-points. Based on this study, 175 mg/m^2^/weekly was recommended for further evaluation in a phase II study. Twenty-three patients were enrolled in their phase II study and found concomitant RT and gemcitabine were well-tolerated with few severe adverse events [67]. Additionally, disease control was observed in both methylated and unmethylated MGMT promoter tumors (91% and 77.5%, respectively).

To date, there is evidence gemcitabine has the ability to cross the blood–brain barrier [68], but some drawbacks include its short plasma half-life, adverse effects related to high drug doses (e.g., myelosuppression, thrombocytopenia, edema), and resistance related to altered expression of nucleoside transporters, kinases, and enzymes [56]. Researchers are currently exploring various delivery strategies for overcoming these limitations (e.g., encapsulation, conjugation, and convention-enhanced delivery) [69,70,71]. For example, Guo et al. surmised gemcitabine coupled to a peripheral benzodiazepine receptor ligand may enhance brain tumor uptake [70]. In their xenograft model, the conjugated agent resulted in a two-fold enhancement in brain tumor selectively compared with gemcitabine alone.

### 5.2. Kinase Inhibitors

#### 5.2.1. Tyrosine Kinase Inhibitors

Tyrosine kinase inhibitors (TKIs) block receptor signaling, inhibiting cell growth and proliferation. Since the approval of imatinib in 2001 for the treatment of chronic myeloid leukemia, there has been an explosion of TKI utilization in multiple types of cancer [72]. TKIs have incredible potential for treating GBM considering their ability to block cell signaling pathways such as EGFR, PDGFR, and VEGF/VEGFR.

**Figure 3:**
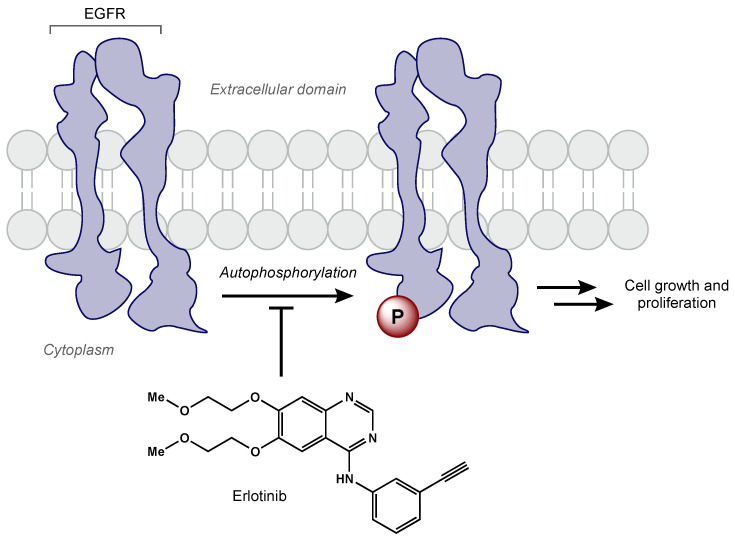
Erlotinib reversibly inhibits EGFR tyrosine kinase activity, which prevents cell growth and proliferation of cancer cells.

EGFR amplification is seen in approximately 40% of GBM cases, correlating with decreased apoptosis, increased cellular proliferation, tumorigenesis, and radioresistance [73,74,75]. Erlotinib is a TKI that has demonstrated activity against the EGFRvIII mutant receptor in preclinical models [76]. Erlotinib is a quinazoline derivative that reversibly inhibits autophosphorylation of EGFR (Figure 3) [77]. Various phase II studies have evaluated the efficacy of erlotinib with concurrent RT and TMZ, but a range of survival and toxicity outcomes have been reported. The first trial included 97 GBM patients who were given erlotinib alone for 1 week followed by concurrent erlotinib, TMZ (75 mg/m^2^ daily), and RT (60 Gy total) [78]. Patients had a median survival time of 15.3 months, but there was no significant benefit compared to RT/TMZ arm of the European Organisation for Research and Treatment of Cancer/National Cancer Institute of Canada trial 26981/22981. Furthermore, molecular subset analysis did not reveal that EGFR amplification was predictive of survival. Another phase II trial included 27 newly diagnosed GBM patients [79]. In this trial, erlotinib was determined to be not efficacious with unacceptable toxicity (grade 3 and 4 toxicities including thrombocytopenia, anemia, lymphopenia, fatigue, and febrile neutropenia). Numerous clinical trials have evaluated other EGFR TKIs (e.g., gefitinib, afatinib) in GBM patients [79,80,81]. Unfortunately, all EGFR TKIs to date have failed to show efficacy in GBM. Researchers hypothesize the lack of efficacy may be due to poor blood–brain barrier penetration, altered signaling pathways, and/or genetic heterogeneity [82].

Recently, preclinical studies have tested osimertinib, a third-generation EGFR TKI, in various GBM cell lines and mice [83]. Liu et al. showed osimertinib inhibited GBM cell growth ten-fold higher than first-generation EGFR inhibitors and prolonged survival in GBM-bearing mice.

#### 5.2.2. mTOR Inhibitors

Rapamycin (mTOR) is a protein kinase that is an important regulator of cell survival and proliferation [84]. mTOR is localized in two distinct multi-protein complexes called mTORC1 and mTORC2 [85]. Previous research efforts have uncovered the critical role of mTOR in GBM pathogenesis [86,87]. Recent studies have shown GSCs can activate the mTOR pathway in microglia, creating an immunosuppressive microenvironment that promotes GBM proliferation [88].

**Figure 4:**
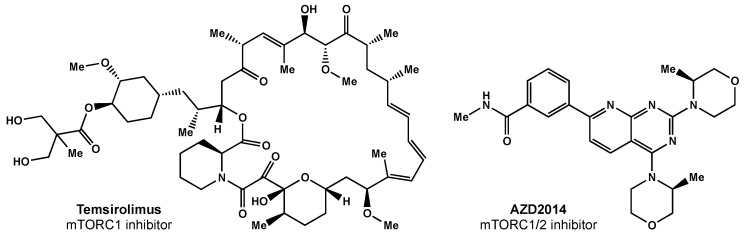
Small molecule inhibitors of mTOR.

Temsirolimus was the first mTORC1 inhibitor investigated in clinical trials (Figure 4). Temsirolimus has been shown to target GICs in preclinical studies, but has failed to demonstrate clinical benefit [89]. Sirolimus, another mTOR inhibitor, also had promising preclinical results, but failed to improve survival, despite being well tolerated [90]. Everolimus, another rapamycin derivative, is a downstream regulator of the EGFR/phosphatidylinositol-3 kinase (PI3K) pathway that has demonstrated radiosensitization in preclinical studies [91]. The North Central Cancer Treatment Group (NCCTG) conducted a phase II trial where weekly everolimus was given concurrently with RT plus TMZ. Ma et al. reported moderate toxicity and survival rates similar to historical phase II trials [92]. The RTOG 0913 trial randomized 171 GBM patients to receive RT with concurrent and adjuvant TMZ with or without daily everolimus (10 mg) [93]. Chinnaiyan and colleagues reported no significant difference in PFS and inferior OS for the patients that received everolimus. There was a significant increase in treatment-related toxicity in patients that received everolimus compared with the control arm; in the experimental arm, there were greater grade 4 and 5 events (30.6% and 11.8%, respectively) than in the control arm (17.9% and 1.3%, respectively).

Researchers surmise that the lack of efficacy may be related to everolimus only selectively inhibiting mTORC1 alone; studies have shown this inhibition can result in increased AKT activation via the activation of mTORC2 [94]. There are ongoing efforts focused on designing a suitable mTORC1/2 inhibitor [95]. AZD2014 is an inhibitor of mTORC1 and mTORC2 (Figure 4) that has shown radiosensitivity in preclinical studies [95] and is being evaluated in a phase I trial (NCT02619864).

### 5.3. Oxygen Mimetics

Conventional RT induces DNA damage via the formation of free radicals generated from the radiolysis of water. Reductants such as glutathione are able to neutralize the radical-induced damage within the cells, but if oxygen is present, this process is prevented, and the damage becomes irreversible. Hypoxic areas of solid tumors greatly hamper the effects of RT, leading researchers to seek oxygen mimetics [96].

Small molecules have been utilized as oxygen mimetics for decades [97] and have historically contained nitro groups that act as electron acceptors [98]. One of the earlier compounds that demonstrated radiosensitizing effects is misonidazole. Although imidazole showed radiosensitizing effects in murine tumors, its lipophilic properties prevented successful translation into clinical trials [99]. Derivatives of misonidazole were tested, and etanidazole had superior hydrophilicity due to the addition of an amide and hydroxyl group [100]. RRx-001 is a dinitro compound originally used as an ingredient in rocket fuel that has demonstrated radiosensitization properties with low toxicity [101]. Currently, RRx-001 is being evaluated in a phase I trial for patients with newly diagnosed glioblastoma (NCT02871843).

Hydrogen peroxide has been explored as a route for enhancing the efficacy of RT [102] and has been evaluated in a phase I/II trial (NCT02757651). Several studies also explored nicotinamide in combination with carbogen breathing in accelerated RT (ARCON) for various tumor types, including laryngeal, bladder, and head and neck [103,104,105,106]. Nicotinamide is a vasoactive agent that decreases perfusion-limited hypoxia, and carbogen (98% oxygen and 2% CO_2_) decreases diffusion-limited hypoxia [107]. Transfusion with red blood cells, in theory, should increase the oxygen supply of tumor cells, but this has failed to demonstrate benefit [108].

### 5.4. Reductive Agents

**Figure 5:**
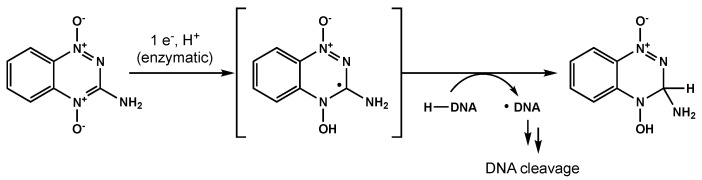
One proposed mechanism for tirapazamine-mediated DNA cleavage under hypoxic conditions.

Bioreductive agents such as quinones and transition metal complexes have garnered attention due to their synergistic effects with RT and their preferential cytotoxicity towards hypoxic cells. Tirapazamine is a pro-drug that can be reduced to a free radical, leading to single- and double-strand DNA breaks under hypoxic environments (Figure 5) [109]. Del Rowe and colleagues conducted a phase II study with RT plus tirapazamine [110]. Although toxicity was acceptable, tirapazamine demonstrated no survival benefit.

An analogue of tirapazamine is SN30000 with more favorable diffusion properties and is currently under development [111,112]. Other analogues such as nimorazole demonstrated efficacy in several trials and are currently used in the treatment of head and neck cancers in Denmark [113].

### 5.5. Histone Deactylase Inhibitors

Histone deacetylases (HDACs) are enzymes that regulate chromatin structure and gene expression via deacetylation of histones and other cytoplasmic and nuclear proteins [114]. Valproic acid, an HDAC inhibitor, has demonstrated increased RT sensitivity in vitro and in vivo. Although the mechanism is unclear, researchers have proposed radiosensitization may be due to the inhibition of chromatin remodeling [115]. Krauze and colleagues conducted a phase II study evaluating the addition of valproic acid to RT plus TMZ [116]. Median OS was 29.6 months (range, 21–63.8 months), PFS was 10.5 months (range, 6.8–51.2 months), and the addition of valproic acid was generally well tolerated. The utilization of valproic acid remains controversial, though, after a pooled analysis found valproic acid at antiepilepsy doses was not associated with improved PFS or OS [117]. Vorinostat is another HDAC inhibitor that has been explored in one phase I/II trial, but failed to meet its primary efficacy end point [118].

### 5.6. Targeting DNA Repair Pathways

Ataxia-telangiectasia-mutated (ATM) serine/threonine protein kinase plays a role in the repair of DNA double-strand breaks [119]. ATM activation is induced within minutes of irradiation, and GSCs are particularly resistant following increased activation of ATM [120,121]. Carruthers et al. demonstrated GSCs display a robust intrinsic phosphor-ATM signal that is further enhanced following irradiation [121]. Other studies have found GBM cell lines and GSCs are radiosensitized by ATM inhibition [122].

**Figure 6:**
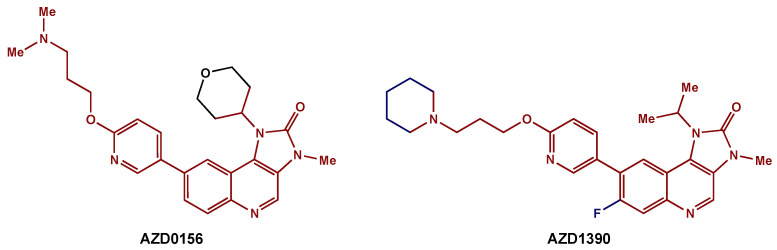
ATM inhibitors: AZD0156 was modified to AZD1390, an orally available compound with greater blood–brain barrier penetrance. The preserved core is highlighted in red.

Recently, medicinal chemists have developed a novel series of ATM inhibitors that demonstrate excellent efficacy and good pharmacokinetic properties [123]. AZD0156 was selected as a suitable candidate for clinical trials (NCT02588105). Further structure–activity relationship lead optimization led to the development of AZD1390, an orally bioavailable inhibitor with greater blood–brain barrier penetrance (Figure 6) [119]. A phase I clinical trial (NCT03423628) is currently recruiting GBM patients for the evaluation of AZD1390 in combination with RT.

### 5.7. Allosteric Modifiers of Hemoglobin

Phenoxyacetic acid compounds were initially utilized as lipid-lowering drugs but later were found to stabilize the T state of hemoglobin [124]. In a phase III trial, efaproxiral, a phenoxyacetic acid analogue, was found to enhance the effect of RT in patients with advanced lung cancer [125]. Kleinberg et al. then surmised GBM patients may benefit from the radio-enhancing effects of efaproxiral because GBM tumors are known to be hypoxic [126] and radioresistant [127]. Although the results were promising, a large dose was needed to reach a therapeutic effect, and long-term dose-related side effects are a concern [128].

### 5.8. Immunotherapy

#### 5.8.1. Anti-Angiogenic Therapy

VEGF inhibitors such as bevacizumab have been explored with the hope of targeting angiogenesis [129]. Chinot and colleagues conducted a phase III trial evaluating the addition of bevacizumab to RT (2 Gy per fraction, total of 60 Gy) plus TMZ (75 mg/m^2^/day for 6 weeks) in patients with newly diagnosed GBM [130]. Although there was increased PFS in the bevacizumab group vs. placebo (10.6 months vs. 6.2 months), there was not a significant difference in OS. Furthermore, there were higher rates of adverse events with bevacizumab than with the placebo. Gilbert et al. also conducted a phase III randomized trial investigating the addition of bevacizumab to RT and TMZ [131]. Their study also demonstrated improved PFS (10.7 months vs. 7.3), although the difference was not significant according to the pre-specified alpha level (*p* < 0.004). The authors also noted a slight increase in adverse events and, over time, a decreased quality of life and neurocognitive function in the bevacizumab group.

#### 5.8.2. Immune Checkpoint Inhibitors

Cancer immunotherapy is based on the concept of immunosurveillance where the immune system can actively detect and eliminate cancer cells, but some tumor cells are able to develop the ability to evade the immune system through immunoediting [132]. Immunoediting is a process where the immune system can both constrain and promote tumor progression [133]. Researchers propose this complex dynamic occurs in three phases: Elimination (the immune system can recognize and kill transformed cells), equilibrium (tumor growth is limited), and escape (edited tumors can grow, unrestrained) [134].

Immunotherapy aims to overcome this immunoresistance with immune checkpoint inhibitors (ICIs) [135]. Immune checkpoints are crucial for self-tolerance, and cancer cells exploit this feature via the upregulation of various pathways (e.g., PD-1/PD-L1, CTLA-4) [136]. Over the past decade, ICIs have revolutionized the treatment of solid tumors and have created renewed excitement within the field of cancer immunotherapy [137].

Although radiation is known to create DNA damage, several studies have suggested the immune system may impact the efficacy of radiation [138]. The exact mechanisms dictating how radiation and the immune system interact are still unclear, but data have revealed CD8 T cells play a key role [139,140]. In theory, combining RT and checkpoint blockage immunotherapy should increase radiosensitization.

Immune checkpoint inhibitors were believed to affect the tumor microenvironment by enhancing the expression of cytokine and chemokine release, which increases immune cell infiltration [141,142]. Anti-PD-1 monoclonal antibodies have had success in the setting of hepatocellular carcinoma, non-small cell lung cancer, renal cell carcinoma, melanoma, and a variety of other solid tumors [136]. Anti-CTLA-4 monoclonal antibodies have also demonstrated a survival benefit for metastatic melanoma [143].

Unfortunately, the addition of ICIs to GBM treatment has led to disappointing initial results. The first major clinical trial evaluating ICIs was CheckMate 143 [144]. In this study, patients with a first recurrence of GBM were treated with anti-PD-1 alone or anti-PD-1 and anti-CTLA-4. Adverse events in the anti-PD-1 plus anti-CTLA-4 arm resulted in discontinuation of the trial, but anti-PD-1 monotherapy was better tolerated. The subsequent CheckMate 143 phase III clinical trial with nivolumab unfortunately failed to improve OS [145]. A small study by Cloughesy and colleagues found pembrolizumab prior to salvage surgery may extend survival [146].

The combination of anti-PD-1 and RT with and without TMZ has also been explored and has been found to be well tolerated. These combinations were then studied in two phase III clinical trials: CheckMate 498 and CheckMate 548. CheckMate 498 evaluated anti-PD-1 as an alternative to TMZ in combination with RT while CheckMate 548 evaluated the addition of anti-PD-1 in addition to TMZ plus RT. In both trials, RT in combination with nivolumab was found to not improve survival [136].

To date, GBM ICI phase III clinical trials have yielded disappointing results. Researchers believe the heterogeneity of GBM tumors may contribute to immunotherapy resistance [147,148]. This tumor heterogeneity makes it difficult to find a singular treatment effective for all GBM patients; therefore, combinatorial strategies are being evaluated (NCT02313272, NCT02311582).

## 6. Recent Preclinical Studies

### 6.1. Purine Metabolism

There is a growing body of literature suggesting purine synthesis contributes to the aggressive nature of GBM [149]. GICs have high rates of de novo purine and pyrimidine synthesis that may contribute to RT resistance [150]. De novo purine synthesis can generate GTP and ATP. GBM preferentially upregulates GTP synthesis, which promotes nucleolar transformation and GBM proliferation [151]. Mycophenolate mofetil (MMF) has been found to inhibit GTP synthesis by blocking the enzyme inosine monophosphate dehydrogenase (IMPDH; Table 1) [152]. Preclinical studies suggest inhibiting GTP synthesis radiosensitizes GBM cells. Because MMF is already FDA-approved, the barrier to clinical translation is low and should be evaluated in patients with GBM.

### 6.2. Metabolic Targeting

Tumor cells predominately utilize glycolysis even in the presence of sufficient oxygen, also known as the Warburg effect [153]. As with many malignant solid tumors, GBM is highly glycolytic and produces lactic acid as a byproduct [154]. Studies have shown tumors with high rates of glycolysis are less responsive to RT and chemotherapy [155]; therefore, researchers have been interested in blocking or reducing glycolytic metabolism as a route for overcoming radioresistance. One study by Shen et al. found treating GBM cells (U87, U251) with a PDK inhibitor and radiation reverses the glycolytic shift [154]. The researchers proposed the inhibitor (dichloroacetate) sensitized GBM cells to radiotherapy by causing G2/M phase cell-cycle arrest (Table 1) [154]. This study suggests that altering the glycolytic metabolism may sensitize GBM to RT [32].

### 6.3. Curcumin

Curcumin has also been explored as a radiosensitizer for GBM [156]. There is evidence that suggests curcumin radiosensitizes tumor cells through various pathways (e.g., modifying activity of RAS-associated proteins, growth factors). Furthermore, curcumin can induce reactive oxygen species generation and inhibit the DNA repair mechanism (Table 1) [157,158]. In a study by Zoi et al., the polyphenol in combination with irradiation (2 to 4 Gy) arrested glioma cells in a synergistic fashion [159].

### 6.4. Hsp90 Inhibitors

Hsp90 is a molecular chaperone that has been associated with protection against radiation-induced cell death [160]. Inhibitors of Hsp90 (e.g., geldanamycin, 17DMAG, radicicol) have been shown to enhance the radiosensitivity of various cell lines (Table 1) [161,162]. Tani and colleagues found *N*-vinylpyrrolidone (NVP)-AUY922 enhanced radiosensitivity in CD133-positive GBM cells [163].

### 6.5. MDM2 Inhibitors

MDM2 has been shown to downregulate p53 activity via ubiquitin-mediated degradation and is amplified or overexpressed in certain GBM patients [164]. MDM2 inhibitors have demonstrated radiosensitizing effects preclinically in other disease sites (e.g., lung cancer, prostate cancer) [165,166], but there are limited data available for GBM. Verreault et al. found MDM2 inhibitor RG7112 reduced tumor growth and increased survival in subcutaneous and orthotopic mouse models (Table 1) [167]. This finding suggests a clinical benefit may be observed in MDM2-amplified GBM patients.

### 6.6. Chimeric Antigen Receptor (CAR) T Cell Therapy

Following the success of CAR T cell therapy in patients with leukemia and lymphoma, researchers have turned their attention towards developing CAR T cells directed toward solid tumors [168]. O’ Rourke et al. conducted a phase I study of autologous CAR T cells targeted to EGFR variant III in patients with GBM [169]. The authors found CART-EGFRvIII cells trafficked to the brain tumors within the first 2 weeks after infusion, but no significant clinical benefit was observed. RT may increase CAR T cell efficacy via alteration of the TME and increasing expression of tumor antigens [170]. Jin et al. found irradiation led to upregulation of CD70 expression on GBM cells and increased CD70-specific CAR T cell tumor cell elimination (Table 1) [171]. Although CAR T therapy has not demonstrated radiosensitizing effects in GBM, exploring potential synergistic interactions between RT and CAR T is an active area of research.

## 7. Conclusions

To date, GBM accounts for a disproportionately high percentage of cancer morbidity and mortality. Extensive research efforts over the past two decades have improved our understanding of the mechanisms driving the treatment resistance seen in GBM. Methods for overcoming radioresistance have been of particular interest, and researchers have been exploring various agents (e.g., oxygen mimetics, kinase inhibitors, immunotherapy agents) as radiosensitizers. Although clinical trials have thus far yielded negative results, recent preclinical results have been promising. Developing small molecules to target GBM-specific features such as increased GTP synthesis or amplification of p53 has the potential for selectively radiosensitizing tumor cells. Additionally, RT has the ability to potentiate the efficacy of immunotherapy, suggesting ICIs and CAR T cell therapy in combination with RT may lead to a synergistic effect. Furthermore, the successful implementation of radiosensitizers in other disease sites have gleaned valuable information and may facilitate the rational design of a GBM radiosensitizer.

## Figures and Tables

**Table 1:** Potential avenues for glioblastoma (GBM) treatment.

Category	Agent(s)	Proposed Mechanism
Purine synthesis inhibitor	Mycophenolate mofetil	GBM upregulates GTP synthesis and mycophenolate mofetil inhibits GTP synthesis
PDK inhibitor	Dichloroacetate	PDK inhibitor that sensitizes GBM cells to RT via G2/M phase cell-cycle arrest.
DNA repair inhibitor	Curcumin	Curcumin radiosensitizes tumor cells and leads to greater G2/M cell-cycle arrest.
Hsp90 inhibitor	Geldanamycin, 17DMAG, radicicol, NVP-AUY922	Targets Hsp90, a chaperone involved in protecting cells against radiation-induced death.
MDM2 inhibitor	RG7112	MDM2 inhibitors increase expression of p53 and may be beneficial in patients with TP53 wildtype and MDM2 amplification.
CAR T cell therapy	CD70 CAR T cells	Targets CD70-expressing GBM tumors and may offset the immunosuppressive effects.
